# Optimizing antimicrobial stewardship in urology: a qualitative study on needs, values and requirements for clinical decision support

**DOI:** 10.1016/j.infpip.2026.100531

**Published:** 2026-03-12

**Authors:** E.M. Engel-Dettmers, L.M.A. Braakman-Jansen, J.E.W.C. van Gemert-Pijnen, R.M. Verdaasdonk

**Affiliations:** a Faculty of Behavioural, Management and Social Sciences, Psychology, Health and Technology, University of Twente, Enschede, the Netherlands; b Department of Clinical Pharmacy, ZGT, Almelo and Hengelo, the Netherlands; c Faculty of Science and Technology, Health Technology Implementation, University of Twente, Enschede, the Netherlands

**Keywords:** Clinical decision support, Needs and requirements, Urology, Antimicrobial stewardship

## Abstract

**Background:**

Urinary tract infections (UTIs) are an important driver of antimicrobial use. Despite antimicrobial stewardship (AMS) initiatives, adherence to treatment guidelines remains poor. Clinical decision support systems (CDSSs) may enhance this, but require alignment with user needs for uptake.

**Aim:**

To explore the experiences, challenges and support needs of urologists in UTI management to identify design requirements for AMS-related CDSSs.

**Methods:**

A qualitative focus group study was conducted among physicians at a Dutch teaching hospital. During two sessions, diagnostic and treatment challenges were explored to elicit motivations for AMS and requirements for decision support. A multi-disciplinary research team used thematic analysis and requirements mapping methods for analysis.

**Findings:**

Participants face multi-faceted challenges in infection treatment, mainly due to missing data and insufficient knowledge, leading to uncertainty in treatment choices. They expressed a need for support, motivated by the desire to improve patient care and increase workflow efficiency, and by the notion to consider antimicrobial resistance (AMR) in prescribing practices. Values for the design of CDSSs are quality of care, efficiency and professional autonomy, translated into requirements for functional properties, user experience and integration into the organizational structure.

**Conclusion:**

Optimizing infection treatment requires an approach that balances effective individual antimicrobial therapy with public health priorities, such as AMR reduction. CDSSs can provide valuable support in this regard, provided that development and implementation are designed with clinical relevance, ease of use, and legal accountability in mind. Addressing the challenges and integrating the requirements from this study into CDSS development helps to create a sustainable and effective AMS strategy.

## Introduction

Urinary tract infections (UTIs) are among the most common bacterial infections worldwide, with a lifetime risk of developing a UTI or interstitial nephritis of 93.7%, and an increasing UTI mortality rate (more than 300,000 deaths in 2021) [1,2]. Due to high relapse rates, frequent hospitalizations, and increasing antimicrobial resistance (AMR), recurrent and complicated UTIs impose a substantial clinical and economic burden [3,4]. The growing prevalence of multi-drug-resistant uropathogens complicates treatment and limits therapeutic options, particularly in more severe cases where ineffective therapy can have serious consequences [5,6].

Given that predominant uropathogens rank among the 10 pathogens making the greatest contribution to AMR-related mortality [3], urologists play a pivotal role in mitigating AMR through appropriate antimicrobial use. To ensure appropriate use, various antimicrobial stewardship (AMS) strategies have been developed [7,8]. AMS teams play a central role in implementing these strategies and guide antimicrobial prescribing, mainly through audit, feedback and education [9,10]. However, the sustainability of these interventions is questionable, as they are labour intensive and, despite efforts, adherence to treatment guidelines remains suboptimal [[11], [12], [13]].

In addition to AMS team interventions, clinical decision support systems (CDSSs) offer promising solutions to enhance antimicrobial prescribing and infection management. CDSSs can generate patient-specific recommendations based on real-time clinical data, guidelines and local AMR patterns. However, the effectiveness of these systems is contingent upon their alignment with clinical workflows and with users' needs and values [[14], [15], [16]]. Consequently, it is imperative to fully understand urologists' needs concerning diagnostics and treatment decisions, along with the barriers they encounter [17,18]. Therefore, this study explored urologists' experiences with infection treatment in both inpatient and outpatient settings. By understanding their clinical decision-making challenges and support needs, design requirements for future decision support technology will be formulated. Through this exploration, this study will aim to help develop a usable technological solution to enhance AMS in urological care at the level of the treating physician rather than the AMS team experts.

## Methods

### Setting and participants

A qualitative focus group study was conducted by a research team consisting of two health sciences researchers, a clinical microbiologist and a hospital pharmacist. Focus groups were convened with urologists and urology residents from a large 700-bed teaching hospital in the Netherlands, which operates two locations. The hospital employs a computerized prescribing order entry system, integrated in an electronic health record (EHR) system, and has a multi-disciplinary AMS team comprising hospital pharmacists, clinical microbiologists, and infectious disease medical specialists. The current activities of the AMS team include the provision of local guidelines, education, and feedback on individual antimicrobial prescriptions.

Participants were recruited by purposive sampling. All urologists and urology residents from the hospital were eligible and were invited to participate by e-mail. As the study's primary focus was to capture the perspective of the intended end-users, and urologists are directly responsible for UTI diagnosis and treatment, other AMS stakeholders were not included at this stage. Participation was voluntary and without financial compensation. The University of Twente Ethics Committee approved the study (Ref. No. 190008), and all participants provided informed consent.

### Study design and data collection

Two one-hour, semi-structured focus groups were conducted to explore challenges and user needs in infection treatment, and to translate these into requirements for AMS-related decision support. EE moderated both sessions, ensuring balanced participation and probing for explanations. Tailored interview guides were created and pilot-tested with a non-participating urologist to ensure clarity.

Focus Group 1 (FG1) explored decision-making challenges, motivations for AMS support, and needs, without predefined conditions. The findings of FG1 were summarized and shared with the attendees for validation. Focus Group 2 (FG2) employed themes from FG1 to select three existing eHealth tools as prompts to discuss desirable functionalities and usability of CDSSs in UTI treatment. Both sessions were audio-recorded and transcribed verbatim.

### Analysis

Transcripts were pseudonymized and coded in Excel (Microsoft Corp., Redmond, WA, USA) by EE. A second researcher (AB) independently coded 25% of the transcripts for triangulation. The final frameworks of themes and subthemes for FG1, and of requirements, attributes and values for FG2, were established through discussion within the research team.

FG1 was thematically analysed following Braun and Clarke's six-step approach [19]. Relevant transcript excerpts were initially coded, iteratively refined and grouped into three predefined themes: (1) AMS decision-making challenges; (2) motivation for support; and (3) needs for AMS decision support. The themes were reviewed and refined through the development of subthemes to capture nuanced insights.

FG2 adopted the requirements development approach proposed by van Velsen *et al.* to translate user needs into actionable design requirements [20]. The analyses involved four stages: (1) initial coding: inductive coding of relevant excerpts to capture user needs and goals; (2) thematic coding: clustering initial codes into overarching attributes that represent core user needs; (3) mapping attributes to values: linking attributes to design values; and (4) converting attributes into design requirements that are suitable for integration into practice.

## Results

### Participants

Six physicians (three male and three female, age 30–62 years) participated in the focus groups. Five physicians participated in FG1, of whom three were urologists with post-fellowship work experience ranging from 11 to 25 years, and two were urology residents. All urologists from FG1 participated in FG2, one resident was new to the study, and one resident from FG1 did not participate in FG2. All physicians had experience in treating UTIs in the in- and outpatient settings of the study hospital.

### FG1: decision-making challenges and support needs for AMS

The analysis of FG1 resulted in 24 distinct codes, which were collated into 10 subthemes, thereby providing a nuanced understanding of each predefined main theme. The thematic framework, encompassing subthemes and codes, is delineated in Table I and will be further elucidated with quotes in the text.

**Table I:** Results from Focus Group 1: decision-making challenges, motivation and user needs for support with antimicrobial stewardship in urology

Theme	Subtheme	Codes	Frequency^a^
1 Current AMS decision-making challenges	1.1 Incomplete or unavailable data	1.1.1 Unavailable data	11
		1.1.2 Incomplete data	3
		1.1.3 Non-useful data	3
	1.2 Invasive data-gathering process	1.2.1 Different information sources	9
		1.2.2 Consulting another specialist	2
	1.3 Collateral medical challenges	1.3.1 Lack of expertise	7
		1.3.2 Intervention by another specialist	4
2 Motivation for AMS support	2.1 Avoidance of AMR	2.1.1 More targeted treatment	10
		2.1.2 Avoid AMR	4
		2.1.3 Infection prevention	1
	2.2 Efficiency	2.2.1 Save time	6
		2.2.2 Save costs	5
	2.3 Improvement of quality of care	2.3.1 Increase patient-friendliness	3
		2.3.2 Increase knowledge	3
3 User needs for AMS decision support	3.1 Automated decision support	3.1.1 Drug choice	5
		3.1.2 Treatment duration	5
		3.1.3 Diagnostics	2
		3.1.4 Drug dose	1
		3.1.5 Predictive data	1
	3.2 Expansion of AMS support	3.2.1 Easy access to AMS team	11
		3.2.2 Expansion of AMS activities	1
	3.3 Clearly presented relevant data	3.3.1 Patient-related data	7
		3.3.2 Infection-related data	3
	3.4 Consider pros and cons of antimicrobial treatment	3.4.1 Alternative treatment options	4
		3.4.2 Effect on microbiome	1

AMS, antimicrobial stewardship; AMR, antimicrobial resistance.

a Number of quotes leading to the relating code.

#### Theme 1: current AMS decision-making challenges

Current decision-making challenges can be divided into three subthemes: (1) incomplete or unavailable data; (2) invasive data gathering; and (3) collateral medical challenges.

Urologists struggle most with incomplete or non-useful data, exemplified by culture results lacking relevant information and limited insight into interactions with other medication used. Moreover, comprehensive patient data beyond the hospital's jurisdiction are unavailable, particularly with regard to information provided by general practitioners (GPs). This leads to the absence of a historical overview of a patient's medication history, thereby complicating the assessment of the patient's response to previous treatments. Incomplete or non-useful data may obscure crucial details about the patient's health journey, thus complicating decision-making and hindering personalized care. Lack of interoperability between systems hampers seamless integration of external data into diagnostic and treatment processes:

*“It also has to do with the various information systems we use here in the hospital and at the GPs. We don't have the authority to access [the EHR] at the GP's office. There is no link with [the microbiology department] for us to be able to view cultures from external sources.” (Urologist P3)*

Furthermore, participants addressed the issue of the invasive data-gathering process. Accessing clinical patient data is often intricate, requiring multiple steps. For instance, urologists are required to liaise proactively with the medical microbiology department when culture information is unavailable, thereby introducing an additional layer to the communication chain:

*“When the information comes by fax, it is scanned. If it's verbal, the secretary has to jot down a note, and whether the note is scanned or it can also be conveyed over the phone, you often end up with incomplete information because they won't delve into all the details. So, yes, I personally find it quite cumbersome, incomplete, and certainly not up to modern standards.” (Urologist P1)*

Finally, the participants encounter collateral medical challenges. As the most recent prescriber becomes the problem owner, cross-speciality consultation is often required. Urologists face uncertainty in circumstances where the clinical relevance and urgency are unclear, such as when prescribing antibiotics that may have cardiac implications. This predicament often necessitates the involvement of other specialists:

*“… patient complexity. Indeed, the use of immunotherapy, renal dysfunction, allergies, and threatened body prosthetics. These are all instances when you find yourself thinking, 'Wait a moment, guys, think along with me for a bit’.” (Urologist P1)*

Beyond technical inefficiency, these data gaps appear to undermine clinicians' sense of diagnostic control and confidence in their therapeutic choices. This highlights how information fragmentation translates into cognitive uncertainty.

#### Theme 2: motivation for support with AMS

Motivation for support with AMS can also be dissected into three subthemes, contributing to: (1) avoidance of AMR; (2) increase in operational efficiency; and (3) enhancement of quality of care.

Avoiding AMR development proves to be an important motivator for the participants, and encompasses the guidance of healthcare providers towards the appropriate use of antimicrobial agents or more targeted treatments. However, their motivation was framed largely in terms of professional and societal responsibility rather than immediate clinical need, suggesting that AMR awareness operates at a conceptual rather than behavioural level:

*“Look, the urgency with the clinical patient suffering from sepsis is that you want to have that patient better as soon as possible. Their health is at risk. The urgency for the outpatient patient is much more its chronic nature. The development of resistance that will occur. And that may be difficult for that patient, but it's also challenging for us, for the community. It's actually very serious, but not immediately for that individual patient.” (Urologist P1)*

Furthermore, decision support would help to save time through increased efficiency, thereby reducing unnecessary expenses and consequently enhancing cost-effectiveness:

*“It would also be helpful for outpatients to quickly know the given treatment is right. It may be less urgent [compared with the clinical setting], but there can still be consequences and costs if we get off to a bad start or have to make changes.” (Urologist P3)*

The third subtheme pertained to the notion that AMS support could enhance the quality of care by facilitating knowledge acquisition and augmenting the patient-friendliness of treatments. This reflects an underlying learning orientation: urologists perceive AMS not merely as regulation, but as an opportunity to expand expertise:

*“We treat a lot of infections, but we are not actually infection experts and certainly not when it becomes a bit more complex. Then we may do it very well at times, and in our opinion we certainly do it very well, but I think, if you are honest, we often just give it a miss.” (Urologist P2)*

#### Theme 3: user needs for AMS decision support

Urologist particularly emphasize specific essential support needs, thereby underscoring the need for advanced tools and resources. For this, four subthemes can be identified: (1) automated decision support; (2) expansion of AMS support; (3) clear presentation of data; and (4) consideration of pros and cons in antimicrobial treatment.

The desire for automated decision support is identified as the key need expressed most often. This tool should play a pivotal role in facilitating diagnostic processes, guiding individual treatment decisions, and integrating considerations for AMR reduction. This emphasis on automation reveals both trust in technology and a wish to offload cognitive burden:

*“You can also submit a notification with a suggestion for a change. [ …. ] That advice immediately takes into account the known cultures. The fact that this link has already been made, that there is an algorithm behind it: in this case, for such and such a patient and such and such bacteria and that renal function, it might be best to use this drug as an alternative. If this does not work, it is best to use this drug. You could call the microbiologist again, but these are decision moments that you can incorporate.” (Urologist P3)*

Interestingly, participants valued human consultation, such as AMS team accessibility, alongside automation, emphasizing that interpersonal support still plays a critical role in legitimizing clinical decisions:

*“Now you are talking about the AMS team. [ …. ] Very accessible, one phone number that we can simply call for these types of questions or is there an e-mail address where we can simply ask a question as a kind of chat? That would also make it a lot easier.” (Urologist P3)*

Urologists also express the need for clear presentation of relevant data, both patient-related and infection-related. This calls for concise, context-rich data displays which demonstrate that usability and interpretability, and not merely data availability, are central to perceived value:

*“It’s the impression that we get patients with recurrent UTIs, who are treated with nitrofurantoin three times and then get ciprofloxacin once and improve. Then I think: were you treated suboptimally the first three times?” (Resident P4)*

Furthermore, urologists seek measures that allow them to consider the pros and cons of antimicrobial treatment thoroughly. It is acknowledged that, in certain cases, abstaining from antimicrobial prescription can be a viable option. In light of this, urologists express the need for a mechanism akin to an emergency brake, providing them with the ability to pause and consider alternative courses of action. This reveals a reflective ethical stance: urologists seek tools that prompt deliberate decision-making rather than enforcing compliance, aligning with AMS principles emphasizing prudent restraint rather than maximal treatment:

*“We have not discussed measures other than antibiotics at all, which should be discussed more often, especially in the outpatient setting. Apart from all those treatments that no longer help at a certain point. People are becoming more and more resistant, or they get too many side effects or they become allergic. There are quite a few more things.” (Urologist P1)*

### FG2: values and requirements for AMS decision support

The expressed needs in FG2 reflected the following three values which urologists prioritized in their approach to infection management: quality of care; efficiency; and professional autonomy. In order to facilitate the development of future decision support tools that align with these values, expressed needs were translated into functional, organizational, and user experience requirements [20]. Table II presents illustrative quotes from FG2, along with their corresponding attributes, leading to the identified values and design requirements.

**Table II:** Results from Focus Group 2: design requirements for antimicrobial stewardship decision support systems with corresponding attributes, values and illustrative quotes

Requirement type	Requirement with illustrative quotes	Attribute^a^	Value
1 Functional	1.1 The system must provide an overview of infection-related data per patient, including culture results, previous treatments, allergies, contraindications, and urinary catheter presence.	Comprehensive overview	Quality of care
	*“I like it when you are in such a tab that you can see in the blink of an eye, in one sheet, where you can look up infection problems. Now, you always have to click through to other things to see what else needs to be done. You have to look up everything.” (Urologist P1)*		
	1.2 The system must generate advice based on guidelines and patient-specific factors (e.g. cultures, previous treatments, allergies, contraindications).	Treatment advice	Quality of care
	*“That there is clinical decision-making behind it. You recognize that this patient has this renal function, this is the known allergy, there were the last cultures, the patient is this ill. You can get that from these parameters, in this case we recommend starting this and than taking cultures here.” (Urologist P3)*		
	*“Advice is issued based on the guidelines, which are the first choices and that it is not too easy to opt for a drug that is actually a third choice, while the first choice is available. That is actually why we are all here, to prevent antimicrobial resistance and to choose the best drug at that time.” (Urologist P1)*		
	1.3 The system must update generated advice when clinical conditions change.	Treatment advice	Quality of care
	*“Suppose you adjust an antibiotic to renal function and renal function is monitored during hospitalization. The patient is septic, recovers and so does the renal function. It can signal that very well … gosh renal function is restored you would now give a higher dose.” (Resident P4)*		
	1.4 The system must allow users to click through to treatment guidelines and past culture results from the infection-related overview.	In-depth declarative information	Quality of care
	*“Suppose you receive a culture and you then decide to start an antibiotic based on that culture, linking that treatment decision, as it were, to that culture result. That you see: that was then given.” (Resident P4)*		
	*“I find it convenient that when you are in such a tab you can click on guidelines. Guidelines to open: sepsis, urinary tract infections, prophylaxis for interventions. Or refer to links/protocols.” (Urologist P1)*		
	1.5 The system must generate reports that allow assessment of multiple patients.	Compare patients	Quality of care
	*“I can imagine that a ward physician would like to check every day “who do I have on antibiotics and who do I have to stop?”” (Urologist P1)*		
	1.6 The system must transparently show how recommendations are generated to allow users to assess their validity.	Treatment advice	Professional autonomy
	*“So to speak, you have a *E-coli* bacteria, you can choose between three antibiotics, but number one is followed by an interaction with the medication that the patient is using, number two is followed by an allergy, so you automatically end up with the third. That is, of course, very easy and you make far fewer mistakes.” (Urologist P1)*		
	1.7 The system must log generated advice and user actions, including when infection-related data is accessed or advice is (not) followed.	Logging of actions	Professional autonomy
	*“You are taking a risk. If you have a system that gives you clear advice and you don't follow it.” (Urologist P1) “Then you have to be able to justify what you did.” (Urologist P2)*		
2 Organizational	2.1 The system must be integrated into electronic medical records to prevent duplication of effort and ensure infection-related data is grouped.	One system	Efficiency
	*“If advice is written in the patient's file, then I think I should take a look at it. That is a way of communicating that is appreciated and that is also read.” (Urologist P3) ….” It is easy and I don't have to click it away. You see it, you can keep reading it.” (Urologist P1) “It will still be there next time and whoever reads the file can see it too.” (Urologist P2)*		
	2.2 The system must interoperable and automatically cluster data for efficient display, minimizing repetitive tasks.	Register once	Efficiency
	*“Now you have to select those patients yourself. Opening such and interface again. The action lies with the physician who has to think about something again.” (Urologist P2)*		
	2.3 The system must generate advice at relevant moments in the workflow.	Minimize obtrusion	Efficiency
	*“[Pop-up signal] That you have to click away. Which is very annoying, because often it also takes a few seconds before you …. Then you have finished your things and then suddenly it appears on the screen. While you think you did already finish.” (Resident P4)*		
	2.4 The system must provide information to support decision-making without over-riding clinical judgement. It should offer an overview and advice while allowing the physician to make the final decision.	Room for professional's view	Professional autonomy
	*“Of course, the patient may have said the day before that it makes him feel sick. That will not always be in the system. That is why advice should always leave a choice.” (Urologist P1)*		
3 User experience	3.1 The system must provide advice that is directly applicable, without requiring extra actions, except in critical situations.	Minimize obtrusion	Efficiency
	*“During a pop-up signal you can't go any further, you can't look at the patient file to see what was the reason I gave gentamicin, you can't click on anything else. So you have to click it away.” (Urologist P2)*		
	3.2 The system must visually emphasize new or critical information using colours or symbols.	Highlight information	Quality of care
	*“if it makes something orange or there is an exclamation mark, then you know there is something to look at.” (Resident P4). For example, with resuscitation policy, there is an exclamation mark that says, take note, this needs to be discussed.” (Resident P6)*		
	3.3 The system must support physician learning by linking patient data with treatment advice and easy guideline access.	Learning tool	Quality of care
	*“I think that you will be able to learn very quickly. Especially if advice comes from the system. So, if you say “hey man, we have a culture, a Proteus culture”, and time and again the system gives the advice to use ciprofloxacin, which you will get very quickly into your system: Proteus that's ciprofloxacin.” (Urologist P1)*		

a Core user need.

#### Functional requirements

Functional requirements focus on technical capabilities, with the primary objective being enhancement of the quality of care. Participants emphasized the need for personalized treatment advice informed by patient data, including allergies, renal function and culture results, integrated with up-to-date guidelines and revised when clinical conditions change. Additionally, urologists require a clickable, patient-specific infection overview, including background information such as treatment guidelines, prior cultures and medications. The system should also link previous treatments to culture results, facilitating physicians to easily find and understand historical treatment choices without having to look in different places. Subsequently, a multi-patient overview was desired for the purposes of quality improvement, AMS meetings and rapid assessments by on-call physicians.

Concerns were also raised. In order to maintain professional autonomy, the system should offer transparent insight into the generation of advice, thus enabling the end-user to assess its validity. This information must be readily accessible for all advice that is generated. Subsequently, the availability of decision support has the potential to engender novel risks, either by following flawed advice or by disregarding appropriate advice. In order to mitigate risks associated with the use of technology, the system must log the generated advice as well as user actions.

This duality, embracing automation while insisting on transparency and control, reflects an intrinsic professional negotiation between safety, accountability and autonomy. It indicates that decision support will only be trusted if its reasoning process is auditable and respects clinical judgement.

#### Organizational requirements

Organizational requirements stipulate the manner in which technology should be integrated into the organizational structure and existing workflows, both for efficiency reasons and to safeguard physician autonomy. Integration is not merely a logistical requirement but a symbolic one: a system embedded in the EHR conveys legitimacy and continuity, while external tools are perceived as peripheral and burdensome. It was asserted by the participants that, in order to circumvent the need to switch between systems, it is imperative that the system is integrated into the prevailing EHR infrastructure. Moreover, they emphasized the importance of the system clustering pertinent data automatically for efficient use, thereby underscoring the need for interoperability. The generation of treatment advice must be timely, and the system must support and not over-ride clinical decision-making. In the event of an unfavourable outcome, it must be clear whether liability is on the physician or on the system.

#### User experience requirements

Requirements for user experiences emphasize smooth interaction between user and system. Participants needed a decision support tool to be unobtrusive, with intuitive navigation and minimal disruption. It was especially important to avoid irritating pop-ups, as they disrupt workflow and have to be clicked away before corrective actions can be taken to improve care. Conversely, participants expressed a preference for passive, unambiguous and actionable advice accompanied by an appropriateness indicator, such as colour coding, that would obviate the need for additional actions.

Participants also considered decision support as a conduit for learning, offering expeditious access to patient data, tailored treatment advice, and easy access to guidelines. The knowledge thus acquired may be employed for the treatment of future patients and the instruction of colleagues. Embedding educational elements within the CDSS design may further enhance user acceptance.

## Discussion

This study provides a qualitative perspective on views and experiences of urologists regarding UTI treatment. It highlights the challenges that urologists face and their needs for decision support. Motivation for support was three-fold: a desire to increase workflow efficiency; a concern to improve patient care; and the notion to consider AMR in prescribing practices. Antimicrobial selection extends beyond infection control, as the increasing prevalence of resistant bacterial strains necessitates more judicious prescribing to safeguard the effectiveness of existing antimicrobials for future generations. While physicians acknowledge AMR as a concern, daily clinical practice frequently prioritizes avoiding undertreatment of individual patients [[21], [22], [23], [24]]. Participants in this study echoed this concern, and regarded decision support as a way to prevent overuse or misuse of antimicrobials, thereby aligning individual care with public health goals, such as AMR reduction.

The primary challenges encountered by participants in this study pertained to missing data and insufficient knowledge, resulting in uncertainty regarding treatment choices. To address these challenges, participants expressed a need for enhanced overview of available information and accessible, context-specific decision support. This support should be based on a combination of patient-related information and up-to-date treatment guidelines. The expressed needs illustrate readiness for the use of decision support technology, and provide a foundation for CDSS development. In the literature, AMS-oriented CDSSs take several forms and are designed for a variety of end-users. CDSSs intended for use by treating physicians do not usually create an overview of available information, and are not able to create context-specific recommendations, or to do so, users frequently need to add patient information manually [14,15,25].

Shapiro Ben David *et al.*, Akhloufi *et al.* and Gaud *et al.* studied CDSSs that better matched the participants' needs by realizing a better contextual fit and EHR integration; however, they still encountered issues with uptake and sustainability [[26], [27], [28]]. Actual use of CDSSs depends upon multiple factors, including the perceived usefulness, credibility and workflow integration [18,[29], [30], [31]]. This underscores why CDSS requirement elicitation procedures should be end-user specific, and should not be limited to designing technology, but should also encompass people and context [20,30,32]. Functional requirements derived from this study focus primarily on improving the quality of care, and demonstrate both trust in, and familiarity with, CDSS utilization. This trust aligns with evidence on CDSS efficacy in the existing literature [25,33,34]. However, the attributes underpinning organizational requirements reflect concerns about CDSS usability in clinical practice, and poor workflow integration is a common cause for reduced CDSS uptake [[27], [28], [29]]. Finally, the emphasis on professional autonomy in this study reflects concerns about responsibility and accountability, also seen by others [35,36]. This specific combination of trust in, and concern about, the potential use of CDSSs in the study findings highlights the importance of an individualized design and implementation process, implying that multiple strategies are needed to engage end-users in order to ensure uptake.

Although the primary objective of CDSS development is to enhance quality of care, implementation can also introduce new risks, including design-induced user errors or flawed algorithmic recommendations. While CDSSs facilitate decision-making, they do not replace clinical judgement, and the onus to exercise decisional autonomy ultimately rests with the physician [37]. The legal and ethical concerns about professional autonomy raised by the study participants thus warrant explicit attention, as existing eHealth development and implementation frameworks pay limited attention to these aspects [[38], [39], [40]].

For this study, focus groups were chosen to encourage open in-depth discussion among participants, allowing for debate and diverse viewpoints. This approach helped to identify a wide range of issues, and contributed to the richness of the thematic analysis. This study also has some limitations. Conducted in a single medical centre, focusing on UTI treatment, this study does not aim for generalizability. Moreover, as all focus group participants were physicians, the perspectives of other stakeholders were not included. Consequently, specific elements of CDSS design, such as technical system integration or organizational impact, remained unaddressed, precluding the formulation of corresponding requirements [20]. These choices were deliberate because contextual insight in the early stages of CDSS development is essential to ensure that the resulting requirements are grounded in workflows, cognitive processes, practical challenges, and perceptions of the intended end-users. Nevertheless, the study provides transferable insights relevant for AMS in other specialties, institutions or healthcare systems, because, as demonstrated, many of the identified needs reflect universal issues in the field of AMS support, and also because this research adds insights about professional autonomy to existing frameworks.

In conclusion, optimizing infection treatment requires a multi-faceted approach that balances effective individual antimicrobial therapy with public health priorities such as AMR reduction. CDSSs can provide valuable support in this regard; however, their success depends on usability, contextual relevance, and respect for professional autonomy. Addressing the identified challenges, and integrating the requirements from this study into future research in other settings and countries, can help successful design and implementation of adaptable, context-sensitive and user-friendly CDSSs.

## CRediT authorship contribution statement

**E.M. Engel-Dettmers:** Writing – review & editing, Writing – original draft, Visualization, Validation, Methodology, Investigation, Formal analysis, Data curation, Conceptualization. **L.M.A. Braakman-Jansen:** Writing – review & editing, Writing – original draft, Visualization, Validation, Methodology, Formal analysis, Conceptualization. **J.E.W.C. van Gemert-Pijnen:** Writing – review & editing, Visualization, Supervision, Methodology, Formal analysis, Conceptualization. **R.M. Verdaasdonk:** Writing – review & editing, Visualization, Supervision, Formal analysis.

## Declaration of generative AI and AI-assisted technologies in the manuscript preparation process

During the preparation of this work, the authors used DeepL in order to improve language and readability. After using this tool, the authors reviewed and edited the content as needed, and take full responsibility for the content of the published article.

## Funding sources

None.

## Conflict of interest statement

None declared.
